# Design of personalized scoliosis braces based on differentiable biomechanics—Synthetic study

**DOI:** 10.3389/fbioe.2022.1014365

**Published:** 2022-11-10

**Authors:** Kateryna Kardash, Christos Koutras, Miguel A. Otaduy

**Affiliations:** Department of Computer Science, Universidad Rey Juan Carlos, Madrid, Spain

**Keywords:** computational design, biomechanical models, differentiable simulation, scoliosis, computational modeling, spine, scoliosis braces

## Abstract

This work describes a computational methodology for the design of braces for adolescent idiopathic scoliosis. The proposed methodology relies on a personalized simulation model of the patient’s trunk, and automatically searches for the brace geometry that optimizes the trade-off between clinical improvement and patient comfort. To do this, we introduce a formulation of differentiable biomechanics of the patient’s trunk, the brace, and their interaction. We design a simulation model that is differentiable with respect to both the deformation state and the brace design parameters, and we show how this differentiable model is used for the efficient update of brace design parameters within a numerical optimization algorithm. To evaluate the proposed methodology, we have obtained trunk models with personalized geometry for five patients of adolescent idiopathic scoliosis, and we have designed Boston-type braces. In a simulation setting, the designed braces improve clinical metrics by 45% on average, under acceptable comfort conditions. In the future, the methodology can be extended beyond synthetic validation, and tested with physical braces on the actual patients.

## 1 Introduction

Scoliosis is a clinical condition marked mostly by a lateral curvature of the spine. Moderate cases of adolescent idiopathic scoliosis (AIS) are typically treated through conservative methods (Karimi and Rabczuk, 2018), which try to naturally correct scoliosis during the growth of the patients. A common conservative treatment is to use orthotic brace structures (Negrini et al., 2022) that transmit forces to the spine and try to correct the existing deformities (Kuroki, 2018; Kaelin, 2020). Such scoliosis braces are designed in a variety of shapes and procedures, but most design methods rely to date on physical experimentation and prototyping. Even though computational strategies have been studied to some extent (Cobetto et al., 2016; Vergari et al., 2016), they are typically limited to evaluating design iterations. The design decisions on the braces are not made by intelligent computer programs; they are instead made by clinical experts, using information from simulation models.

In our work, we try to answer if brace design can be further automated, devising an intelligent computer program that can automatically explore the design space of a brace, and select optimal design parameters. The development of such a brace design solution faces two important research challenges. First, it requires a personalized biomechanical model of brace-trunk interaction. Using this biomechanical model, it is possible to predict trunk deformations as a function of changes to the brace design. This simulation is key for computer evaluation of design metrics, such as a clinical objective and a comfort objective. Second, the biomechanical model must be embedded within a numerical optimization solver, which will automatically search for brace design parameters. State-of-the-art optimization methods use gradients to efficiently and robustly search for optimal parameters; therefore, the biomechanics formulation must allow for the computation of gradients with respect to design parameters.

In this work, we propose a differentiable biomechanics model for the optimization of scoliosis brace designs. By formulating biomechanics simulation as a differentiable function, we can embed the simulation model within advanced optimization procedures, and automatically achieve effective brace designs, with flexible definition of design goal metrics.

The first major component of our approach, presented in Section 3, is a predictive model of the passive biomechanics of the trunk. We put the focus on modeling biomechanics elements that affect the deformation of the thoracolumbar spine under forces produced by a scoliosis brace. To this end, we largely rely on previous work for the development of the trunk model (Koutras et al., 2021) and personalization of its geometry (Koutras et al., 2022) (mechanical personalization is left as future work).

The second major component of our approach, presented in Section 4, is an optimization algorithm that relies on differentiable biomechanics. We show that differentiable biomechanics can be efficiently formulated by implicitly linearizing the equilibrium constraints of a biomechanics simulation engine, and we provide insight into the design of all elements of the biomechanics model to ensure efficient and robust differentiation.

We have tested our personalized brace design approach on a cohort of 5 (potential) patients of AIS. For each patient, we personalize the trunk model, initialize a Boston brace design (Périé et al., 2003), and then optimize this design using the differentiable biomechanics formulation. The results of our experiments show that a fully automated algorithm manages to reduce Cobb angle by an average of 7.8°, while keeping brace forces within a comfortable range of 220 N. To date, we have limited our experiments to a synthetic study, considering only simulated brace-trunk interactions. Our work demonstrates the feasibility of the approach, but real-world application requires further iterating other aspects of the brace design, as well as validating the clinical and comfort objectives before letting actual patients wear the resulting braces.

## 2 Related work

### 2.1 Trunk modeling and parameter estimation

Designing a personalized biomechanical model entails two tasks: fitting the morphology and connections of anatomical elements to those of the patient, and parameterizing the mechanical models to match the response of the patient’s body. Biomechanical modeling of the spine has received a lot of attention, with popular approaches largely divided into two categories. One category follows the Finite Element Method (FEM, please see (Wang et al., 2014) for a survey of methods); the other category uses a simpler but more efficient solution based on multi-body models.

In terms of FEM models, many approaches have been developed for the lumbar (Xu et al., 2017; Finley et al., 2018; Dong et al., 2020), thoracic (Aroeira et al., 2017; Aroeira et al., 2018) or the cervical spine (Lasswell et al., 2017). Furthermore, Dicko et al., (2015) developed a hybrid lumbar spine model containing rigid bodies, FEM and contact mechanics, while Clin et al., (2011) developed a novel method to include gravitational forces in an FE model. While these methods are potentially accurate, they require careful estimation of model parameters for personalized design applications.

FE models of the trunk have been coupled to brace models and patient geometry for personalized brace design in the context of AIS (Gignac et al., 2000; Liao et al., 2007; Nie et al., 2009). Some studies include the evaluation of the effectiveness of these techniques on large cohorts of patients (Cobetto et al., 2017; Vergari et al., 2020; Guy et al., 2021).

In terms of multi-body models, de Zee et al., (2007) made a generic detailed rigid-body model of the lumbar spine. Bayoglu et al., (2019) developed a multi-body muscoloskeletal model of the human spine in order to study the spinal loads. Raabe and Chaudhari, (2016) investigated the jogging biomechanics using a full-body spine model developed in OpenSim, an open-source musculoskeletal simulation software. Le Navéaux et al., (2016) developed biomechanical models based on multi-body dynamics to analyze the effects of implant density and distribution on curve correction and the resulting forces on the vertebrae. Ignasiak et al., 2016 predicted the dynamic spinal loading using a multi-body thoracolumbar spine model with articulated rib cage.

In the category of multi-body models, a strong effort has been devoted to finding accurate simplifications of the models and designing parameter estimation techniques. In our design and parameterization of a spine-and-trunk model, we borrow insight and design choices from this collection of work.

Panjabi et al., (1994) studied the mechanical behavior of the human lumbar and lumbosacral spine as shown by three dimensional load-displacement curves. Panjabi et al., (1976) estimated the rotational stiffness coefficients of the thoracic spine from experiments. Bisschop et al., (2012) and Panjabi et al., (1976) found the translational stiffness coefficients of the thoracic spine through experimental studies. Moroney et al., (1988) estimated the load displacement properties of the cervical spine from experiments. Andriacchi et al., (1974) estimated the stiffness coefficients of the elastic properties of the rib cage through simulations. Wilke et al., (2017) examined the flexibility of every thoracic spinal segment in an *in vitro* experiment. Liebsch et al., (2017); Liebsch et al., (2019) investigated the kinematic and stiffness properties of the thoracic spine and the rib cage through experimental studies.

Moreover, many studies tried to estimate the mechanical parameters of the soft tissue in the human’s body. Choi and Zheng, (2005) estimated Young’s modulus and Poisson’s ratio of soft tissue from indentation using two different-sized indentors in a finite element analysis. Song et al., (2006) studied the elasticity of the living abdominal wall in laparoscopic surgery. Hostettler et al., (2010) measured the Bulk modulus and volume variation of the liver and the kidneys *in vivo*. McKee et al., (2011) compared the reported values of Young’s modulus obtained from indentation and tensile deformations of soft biological tissues.

### 2.2 Optimization-based shape design

As mentioned earlier, some works have considered computational optimization of scoliosis braces leveraging simulation models of brace-trunk interaction (Gignac et al., 2000; Liao et al., 2007; Nie et al., 2009). These works suffer strong limitations though. On one hand, the biomechanics models they use are limited, e.g., some limit brace interaction to point forces applied on the skin surface (Gignac et al., 2000). On the other hand, the optimization solvers require complex simulation of trunk biomechanics for every small optimization step, which turns into large inefficiencies.

The computational approach explored in this work considers tight connection between the optimization formulation and the biomechanics engine, to design efficient search methods. This approach is popular in the fields of parameter estimation and computational fabrication, and it relies on implicit differentiation of the biomechanics model. For instance, the work of Miguel et al., (2012) used implicit differentiation of a cloth model to execute efficient parameter estimation of cloth simulation models, and the works of Pérez et al., (2015); Pérez et al., (2017) used implicit differentiation of rod meshes to explore shape designs later produced through 3D printing. Recently, differentiable simulation methodologies have also seen success in robotics. The applications include the optimization of control policies (Du et al., 2021; Murthy et al., 2021), or even the design of sensor networks (Tapia et al., 2020).

It is important to note that computational optimization methods have been explored for various objects, very different from braces, but the methodologies could be applicable. Montes et al., (2020) recently developed a design method for tight clothing that considers design and comfort metrics, and accounts for simulation models of both clothing and the body. Zhao et al., (2021) recently developed a similar method to design supporting surfaces for the human body, accounting for metrics that optimize ergonomics.

## 3 Trunk and brace simulation model

In this section, we describe the biomechanical model of the torso, the model and parameterization of the brace, and the interaction between both. We put all these elements together in a biomechanics simulation engine that, given a brace geometry as input, outputs the deformation of the trunk and the forces between trunk and brace. We borrow the biomechanical model of the trunk from the work of Koutras et al., (2021), and we refer to their publication for details.

In the definition of the biomechanical model of the trunk and brace, we seek a formulation that can be differentiated, to be efficiently embedded within a design optimization approach. To this end, we model all biomechanical components using smooth energy potentials.

### 3.1 Biomechanical model of the trunk

The purpose of the trunk simulation model is to predict the deformation of the spine under the action of a scoliosis brace. To this end, we characterize the trunk as a multi-body system, consisting of articulated rigid bodies to model the skeleton, and surrounding soft tissue to model the skin, muscles and organs. In particular, and following the rationale of (Koutras et al., 2021), we consider the trunk model passive, without muscle activation. While the exact instantaneous trunk deformation depends on the instantaneous muscle activity, we consider a passive trunk model as an approximate average of muscle activity conditions.

We group all degrees of freedom (DoFs) of the trunk in a large vector **x**_trunk_. This vector includes: 1) translation and rotation of all bones in the trunk skeleton (the rotation is parameterized using incremental axis angle (Taylor and Kriegman, 1994)); and 2) nodes of a tetrahedral mesh of the soft tissue. The trunk skeleton includes the bones relevant for predicting the deformation of the lumbar and thoracic spine, which are: all lumbar and thoracic vertebrae, the sternum, the rib cage, and the pelvis. The soft-tissue mesh is constructed from the surface of the trunk between neck and waist, and clipped at the shoulders. We provide as input this surface as a triangle mesh, together with sample points on the surfaces of the bones, and we produce a conformal tetrahedral mesh (Si, 2015). Introducing mesh nodes on the surfaces of bones facilitates the coupling between skeleton and soft tissue. We rigidly couple these nodes to the bones, and we remove them from the DoF vector **x**_trunk_.

The mechanics of the trunk are modeled as a sum of energy terms parameterized by the DoFs **x**_trunk_. The energy terms include compliant joint models between skeletal bones *W*_joints_ (**x**_trunk_), and the soft-tissue internal energy *W*_tissue_ (**x**_trunk_). We describe the joint energies *W*_joints_ as 6-DoF joints between pairs of bones, acting on both their translation and rotation. By tuning the stiffness of each constraint axis, we effectively model different types of joints and their anisotropy. We describe the soft-tissue energy *W*_trunk_ using a Neo-Hookean strain energy density model, integrated on the volume of the tetrahedral mesh using a finite-element formulation. As mentioned above, we refer to (Koutras et al., 2021) for details on the biomechanical model of the trunk, such as the definition of the energy terms.

In our study, we use the trunk model to simulate the biomechanical response of different subjects suffering from AIS. To adapt the model to each subject, we start from a template geometry corresponding to a healthy (non-scoliotic) adolescent female, and then we apply the morphing method in (Koutras et al., 2022), using as input low-dose radiographs of the subjects. Full personalization would also require estimating subject-specific mechanical parameters, but in this synthetic study we limit ourselves to the default mechanical parameters listed in (Koutras et al., 2021).

### 3.2 Model and parameterization of the brace

There are many different types of scoliosis braces. Designing a computational solution that explores the different brace types is a complex task, as the design space is not continuous. Therefore, for our solution we focus on one particular type of brace, specifically the classic Boston brace (Périé et al., 2003), which concentrates brace-trunk forces on three locations. One of these locations corresponds to the location of peak curvature in the spine, and the other two locations are above and below, to produce counteracting forces and a net torque that rotates the spine in a corrective direction. Note that more modern variants of the Boston brace consider also other design principles, but we leave such extensions for future work.

**FIGURE 1:**
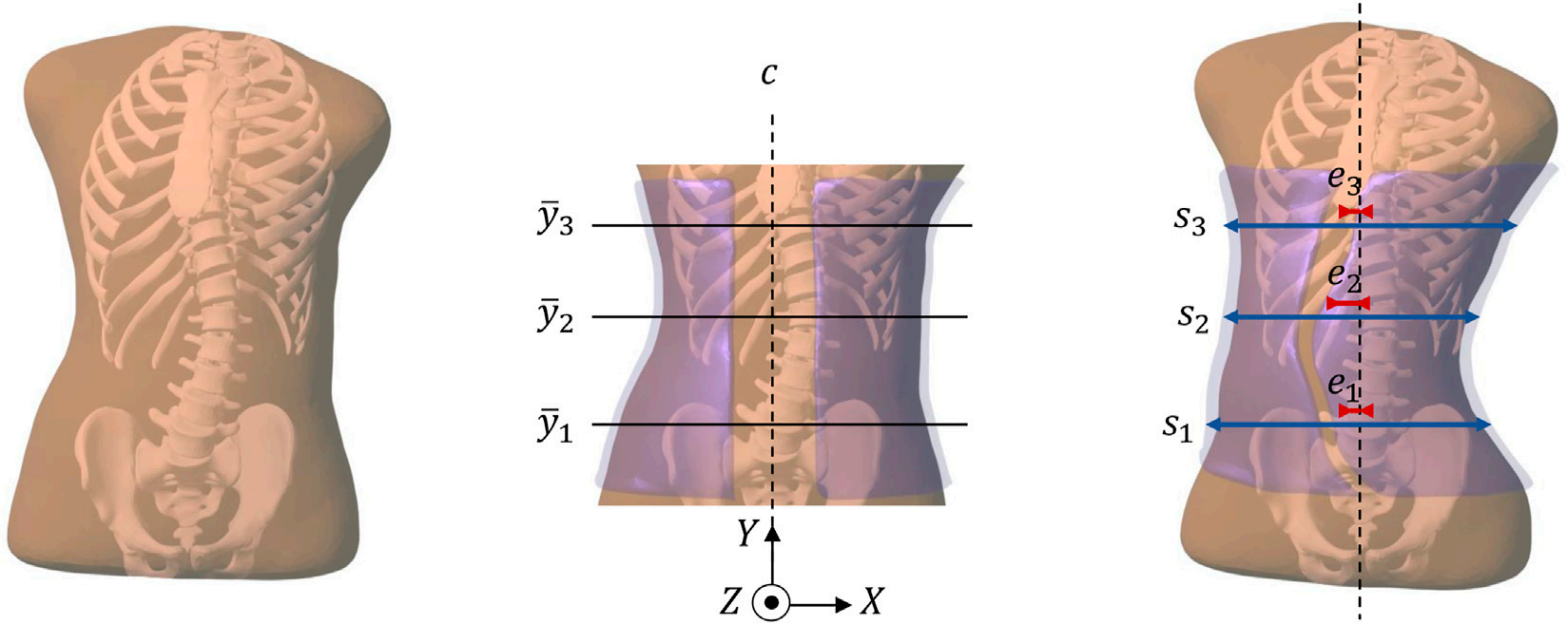
This figure summarizes the pipeline of our computational brace design methodology. From left to right: (i) Starting from radiographs of a patient, we obtain a personalized simulation model of the trunk (including the spine, the rib cage, and the surrounding soft tissue). (ii) We initialize a brace geometry (in semi-transparent purple) by trimming and slightly offsetting the patient´s skin. In this initial brace geometry, we identify the frontal center line *c*, and three heights ${\bar{y}}_{1},{\bar{y}}_{2},{\bar{y}}_{3}$ where we focus the contact forces between brace and trunk. (iii) We automatically optimize the eccentricity *e* and horizontal scale *s* at the three heights. As a result, the brace deforms the full trunk, and produces a correction of the spine curvature.

We describe the geometry of the Boston brace as a parametric surface S(p) , which depends on a set of design parameters **p**. Figure 1 shows how we model and parameterize the brace geometry. We start by initializing a reference geometry $\bar{S}$ , by copying the geometry of the trunk surface, clipped at the waist and under the armpits. This clipped surface covers the scoliotic portion of the spine. Computer optimization of the brace shape requires a compact parameterization of its geometry. There are many possible approaches to define this parameterization, such as the use of control points and spline surface definitions. In our prototype solution, we rely heavily on the 3-point design methodology of Boston braces, and we parameterize the brace based on *X*-axis scale *s* and eccentricity *e* with respect to the *X*-axis center line *c* at three different heights. Our choice of design parameters is aimed at maximizing impact on the lateral flexion of the spine; however, since the trunk is modeled fully in 3D and the axial rotation of the spine is not constrained, the brace will also produce the physically expected axial rotation. To mimic the 3-point methodology, we pick the height of the maximum spine curvature, and two heights above and below this location. We linearly interpolate scale and eccentricity values along the height of the brace. Formally, given an initial brace point $\left(\bar{x},\bar{y},\bar{z}\right)\in \bar{S}$ , the design transformation returns a point $\left(x=s\left(\bar{y}\right)\cdot \left(\bar{x}-c\right)+c+e\left(\bar{y}\right),\;y=\bar{y},\;z=\bar{z}\right)$ .

In addition to the design transformation, the initial shape of the brace is transformed to provide pressure on the trunk, and thus forces on the spine that produce an incremental correction. This produces the actual brace surface $S\left(\bar{S},p,x_{\text{brace}}\right)$ , which depends on the initial brace geometry $\bar{S}$ , the design parameters **p**, and some brace transformation DoFs **x**_brace_. Following common designs of Boston braces, we split the brace surface vertically in two-halves. Each of these halves is modeled mechanically as a rigid body, i.e., we ignore the deformation of the brace. On the back of the brace, we connect the two-halves using a vertical hinge joint. With the rigid transformation of one-half of the brace, and the angle of the hinge joint, we define the DoFs of the brace **x**_brace_ in the simulation. On the front of the brace, we connect the two-halves using a strap. This strap has zero rest-length and a stiffness *k*_*strap*_. Then, given strap connections *x*_left_ and *x*_right_, the strap is modeled mechanically as a simple spring with energy

$$W_{\text{strap}}=\frac{1}{2}\;k_{\text{strap}}\;\|x_{\text{left}}\left(x_{\text{brace}}\right)-x_{\text{right}}\left(x_{\text{brace}}\right){\|}^{2}.$$ (1)

### 3.3 Contact and regularization

To model the interaction between the brace and the trunk, we use a potential-based contact model. An alternative would be to solve contact with hard constraints, but the differentiation of hard constraints is considerably more expensive (Liang et al., 2019). Among potential-based contact models, some recent methods propose highly nonlinear potentials to strongly enforce non-penetration (Li et al., 2020). However, we opt for a quadratic potential to improve the convergence of the design optimization solver.

Given a deformation of the trunk defined by its DoFs *x*_trunk_, we define a distance field of its surface, *ϕ*_trunk_ (*x*, **x**_trunk_), *x* ∈ R^3^. The distance is positive outside the trunk and negative inside. Based on this distance field, we define a potential energy that penalizes interpenetration between the brace surface S and the trunk:

$$W_{\text{contact}}=\int_{\bar{S}}\;\frac{1}{2}\;k_{\text{contact}}\;\text{neg}{\left(\phi_{\text{trunk}}\left(S\left(\bar{S},p,x_{\text{brace}}\right),x_{\text{trunk}}\right)\right)}^{2}\;d\bar{S},\;\text{neg}\left(r\right)=\min\left(r,0\right).$$ (2)

We approximate the integral through finite-element integration on the triangular surface elements of the brace surface $\bar{S}$ .

In our contact model, we do not account for friction between the brace and the trunk. Friction indeed exists in the real world when the trunk moves, but it is always possible to adjust the brace and let skin relax to remove such friction. Therefore, we are interested in modeling frictionless interaction.

This said, friction plays another important role in simulation, as it ensures that the trunk cannot slip through the brace. We replace this effect with a small regularization term that tries to maintain the brace in place, by penalizing the norm of the brace DoFs.

$$W_{\text{reg}}=\frac{1}{2}\;k_{\text{reg}}\;\|x_{\text{brace}}{\|}^{2}.$$ (3)

### 3.4 Simulation engine

Based on the various energy terms defined in the previous sections, the total energy of the trunk-brace system can be computed as:

$$W=W_{\text{joints}}+W_{\text{tissue}}+W_{\text{strap}}+W_{\text{contact}}+W_{\text{reg}}.$$ (4)

Given a set of brace design parameters **p**, we wish to predict the deformation of the trunk produced by the brace. We do this by solving a static equilibrium problem. For convenience, we concatenate all DoFs in one large vector $x=\left(x_{\text{brace}},x_{\text{trunk}}\right)$ . Then, we can express the computation of the trunk and brace configuration as the following minimization of the total energy:

$$x\left(p\right)=\arg\underset{x}{\min}W\left(p,x\right).$$ (5)

At the configuration of minimum energy, the biomechanics satisfy static equilibrium, i.e., zero net energy gradient or zero net force everywhere in the brace-trunk system:

$$-{\frac{\partial W\left(p,x\right)}{\partial x}}^{T}=f\left(p,x\right)=0.$$ (6)

Note that the forces on the DoFs, **f**, are simply the (negative) gradient of the energy. Static equilibrium is a direct consequence of the optimality conditions of the optimization. We can also regard these optimality conditions as *biomechanics constraints*.

To solve the optimization (Eq. 5), we use Newton’s method with line search (Nocedal and Wright, 2006). On every Newton iteration, we compute the Jacobian of the forces, i.e., the (negative) energy Hessian, $\frac{\partial f}{\partial x}=-\frac{\partial^{2}W}{\partial x^{2}}$ , we solve a linear system for the change in the DoFs, and we take a safe step that minimizes the energy. Our biomechanics model is implemented in C++ in SOFA (Faure et al., 2012), which allows to implement energies, forces, and force Jacobians in a flexible and efficient way. However, we have built the Newton solver on Python, with bindings to the SOFA model.

## 4 Brace optimization

In this section, we describe the formulation of an optimization problem for brace design. We start with the formulation of a design objective function, and we continue with efficient computation of the objective gradient using our differentiable biomechanics formulation.

### 4.1 Design optimization problem

To guide the change of the brace shape, we need a merit function that quantifies the impact on the spine from a clinical standpoint. In our approach, we use the Cobb angle (Safari et al., 2019) to characterize the severity of scoliosis. More complex, multi-objective merit functions are left for future work, but are briefly discussed in Section 6. The Cobb angle measures the largest angle between the superior end plate of a vertebra and the inferior end plate of some other vertebra, thus characterizing the maximum bending of the spine. The Cobb angle Ψ(**x**_trunk_) can be computed from the DoFs of the vertebrae embedded in **x**_trunk_. Then, our clinical merit function is:

$$L_{\text{Cobb}}={\left(\frac{\Psi \left(x_{\text{trunk}}\right)}{\Psi \left(x_{\text{trunk},0}\right)}\right)}^{2}.$$ (7)

Note that the metric is normalized by the initial Cobb angle $\Psi \left(x_{\text{trunk},0}\right.$ , to simplify weighing of the metric across subjects.

However, optimizing the Cobb angle might produce braces that are uncomfortable or even impossible to wear. Therefore, we also add the total force applied by the brace on the trunk as a comfort function, penalizing designs that would be too uncomfortable. We obtain per-point forces by differentiating the penalty potential in (Eq. 2), we compute its squared norm, and we integrate the result over the surface of the brace:

$$L_{\text{contact}}=\int_{\bar{S}}\;k_{\text{contact}}^{2}\;\text{neg}{\left(\phi_{\text{trunk}}\left(S\left(\bar{S},p,x_{\text{brace}}\right),x_{\text{trunk}}\right)\right)}^{2}\;d\bar{S}=2\;k_{\text{contact}}\;W_{\text{contact}}.$$ (8)

Adding the clinical metric (Eq. 7) and the comfort metric (Eq. 8), we obtain the full objective function of the brace design problem:

$$L=L_{\text{Cobb}}+\lambda \;L_{\text{contact}},$$ (9)

where *λ* is the relative weight of the two components of the objective function. A proper weight adjustment *λ* of the clinical and comfort metrics produces designs that are both effective and comfortable to wear. We consider a Cobb angle of 75% as a sufficient objective, and a force of 250 N as a reasonable contact force. Then, a value of *λ* = 9*e* ^−6^ assigns equal weight to both the clinical and comfort metrics.

### 4.2 Optimization algorithm

Given the brace design objective L in (Eq. 9), the biomechanics energy *W* in (Eq. 4), the DoFs of the trunk and the brace **x**, and the parameterization of the brace geometry **p**, the computation of the brace design can formally be expressed as finding the brace parameters that minimize the design objective, subject to biomechanical constraints:

$$p=\arg\underset{p}{\min}L\left(p,x\right),\;\text{s.t. }f\left(p,x\right)=0.$$ (10)

We rely on numerical optimization methods to automatically search for brace geometries that produce optimal designs. In our implementation, we choose the bounded L-BFGS optimization algorithm (Nocedal and Wright, 2006), as it offers a good balance between speed and convergence. L-BFGS requires the evaluation of the gradient of the objective function with respect to the design parameters, i.e., $\frac{\partial L}{\partial p}$ .

The challenge in the evaluation of this gradient is that a change of design parameters **p** affects the DoFs **x** in a complex way, through the equilibrium condition (Eq. 6). Due to this complexity, a common approach to evaluate $\frac{\partial L}{\partial p}$ is to use finite differences. This approach requires solving the static equilibrium condition for an incremental change to each of the design parameters. Therefore, it can be formally expressed as:

$$\frac{\partial L}{\partial p_{i}}=\frac{L\left(p+\varepsilon \;e_{i},\;\arg\min_{x}W\left(p+\varepsilon \;e_{i},x\right)\right)-L\left(p,\;\arg\min_{x}W\left(p,x\right)\right)}{\varepsilon },$$ (11)

where **e**_*i*_ is a unit vector in the direction of the *i*th parameter, and *ɛ* is a small value. For small parameter sets, finite-differences may be computationally effective, but their cost grows linearly with the number of design parameters, and they soon become the bottleneck of the optimization.

### 4.3 Differentiable biomechanics

Instead of using finite differences, we leverage our differentiable biomechanics formulation to compute the gradient of DoFs with respect to design parameters, $\frac{\partial x}{\partial p}$ , and use this to evaluate the gradient of the objective function.

With the definition of the objective function (Eq. 9), we note that the clinical metric $L_{\text{Cobb}}$ depends only on the DoFs **x**, while the comfort metric $L_{\text{contact}}$ depends on the DoFs but also directly on the brace design parameters **p**. Then, the full objective gradient can be written as:

$$\frac{\partial L}{\partial p}=\lambda \;\frac{\partial L_{\text{contact}}}{\partial p}+\left(\frac{\partial L_{\text{Cobb}}}{\partial x}+\lambda \;\frac{\partial L_{\text{contact}}}{\partial x}\right)\;\frac{\partial x}{\partial p}.$$ (12)

Thanks to the implicit function theorem (IFT), the biomechanics equilibrum constraints (Eq. 6) must be satisfied for small changes to the DoFs and the design parameters. Then, we can differentiate those constraints to obtain:

$$\frac{\partial f}{\partial p}+\frac{\partial f}{\partial x}\;\frac{\partial x}{\partial p}=0\;\;\;\;\to \;\;\;\;\frac{\partial x}{\partial p}=-{\frac{\partial f}{\partial x}}^{-1}\;\frac{\partial f}{\partial p}.$$ (13)

IFT provides an implicit definition of the Jacobian of the DoFs with respect to design parameters, such that the equilibrium constraints remain satisfied.

We leverage this Jacobian to differentiate the objective (Eq. 9) analytically, and substitute it in (Eq. 12) to obtain:

$$\frac{\partial L}{\partial p}=\lambda \;\frac{\partial L_{\text{contact}}}{\partial p}-\left(\frac{\partial L_{\text{Cobb}}}{\partial x}+\lambda \;\frac{\partial L_{\text{contact}}}{\partial x}\right)\;{\frac{\partial f}{\partial x}}^{-1}\;\frac{\partial f}{\partial p}.$$ (14)

The evaluation of the gradient (Eq. 14) requires substituting the implicit derivative (Eq. 13). In practice, however, this is done using the adjoint method:

$$\frac{\partial L}{\partial p}=\lambda \;\frac{\partial L_{\text{contact}}}{\partial p}-u^{T}\;\frac{\partial f}{\partial p},\;\text{with }\frac{\partial f}{\partial x}\;u={\left(\frac{\partial L_{\text{Cobb}}}{\partial x}+\lambda \;\frac{\partial L_{\text{contact}}}{\partial x}\right)}^{T}.$$ (15)

On each step of L-BFGS, given a new tentative set of brace parameters **p**, we solve the static equilibrium (Eq. 5) to obtain the trunk and brace DoFs. Then, we solve for the adjoint **u** in (Eq. 15), and we evaluate the full objective gradient.

### 4.4 Implementation details

Our optimization is built in Python, using the L-BFGS solver in scipy. As mentioned in Section 3.4, we use SOFA for the biomechanics, and then we build the full gradient in Python and we feed it to the L-BFGS solver. By defining smooth biomechanics functions with respect to both DoFs **x** and design parameters **p**, we can efficiently evaluate the objective gradient $\frac{\partial L}{\partial p}$ . Next, we provide some details about the computation of the derivative terms in (Eq. 15).

The Jacobian of forces $\frac{\partial f}{\partial x}$ is already used by the Newton-type solver of biomechanics simulation (see Section 3.4), and it requires all energy terms to be twice-differentiable. This condition is the default for most energy terms, including *W*_joints_, *W*_tissue_, *W*_strap_, and our ad-hoc term *W*_reg_. In Section 3.3, we also pay special care to design a twice-differentiable contact model *W*_contact_.

The gradient of the comfort metric with respect to the DoFs can be obtained trivially from the contact forces. From (Eq. 8), we derive that $\frac{\partial L_{\text{contact}}}{\partial x}=2\;k_{\text{contact}}\;\frac{\partial W_{\text{contact}}}{\partial x}=-2\;k_{\text{contact}}\;f_{\text{contact}}$ .

The gradient of the comfort metric with respect to the brace parameters does not differ much from the contact forces. It can be computed as $\frac{\partial L_{\text{contact}}}{\partial p}=2\;k_{\text{contact}}\;\int_{\bar{S}}\frac{\partial W_{\text{contact}}}{\partial S}\;\frac{\partial S}{\partial p}\;d\bar{S}$ . $\frac{\partial W_{\text{contact}}}{\partial S}$ represents per-point contact forces on the brace, which are then multiplied through the chain rule by the gradient of the transformed brace with respect to the design parameters, $\frac{\partial S}{\partial p}$ . This last gradient can be easily obtained from the definition of the brace parameterization in Section 3.2.

The most complex term is probably the Jacobian of forces with respect to brace parameters, $\frac{\partial f}{\partial p}$ . The forces affected directly by the parameters are the contact forces (on both the brace and the trunk) and the strap forces. Similar to the derivation of $\frac{\partial L_{\text{contact}}}{\partial p}$ above, the Jacobian of contact forces can be obtained as the chain rule of per-surface-point force Jacobians and the gradient of the transformed brace surface with respect to design parameters, i.e., $\frac{\partial f_{\text{contact}}}{\partial p}=\int_{\bar{S}}\frac{\partial f_{\text{contact}}}{\partial S}\;\frac{\partial S}{\partial p}\;d\bar{S}$ . The Jacobian of the strap force, $\frac{\partial f_{\text{strap}}}{\partial p}$ , is simple, as it just involves two points on the surface of the brace, as shown in (Eq. 1).

The last necessary term is the gradient of the clinical metric with respect to the DoFs, $\frac{\partial L_{\text{Cobb}}}{\partial x}$ . To this end, within each iteration of the optimization, we consider that the vertebrae defining the Cobb angle do not change. Then, the expression of Cobb angle becomes a smooth and differentiable formula.

The IFT applied in (Eq. 13) to implicitly define the derivative of DoFs with respect to design parameters is only valid when the biomechanics constraints (Eq. 6) are satisfied. For this reason, the static equilibrium is solved on each L-BFGS iteration. We have experimented with the accuracy required by the static equilibrium solve, which in turn affects the accuracy of $\frac{\partial x}{\partial p}$ . We have concluded that a threshold of 1 mN is necessary to this end. However, this does not affect performance much. We have observed that, in practice, the solver leverages the expected quadratic convergence of Newton’s method once the residual is small.

## 5 Experiments

This section describes the cases that were evaluated in our study. We start with a description of the patient data, and then we provide an analysis of performance of the brace design methodology. As discussed in the introduction, to date we have limited our experiments to a synthetic study, and we evaluate the performance of brace designs only on simulation models of the patients.

### 5.1 Test cohort

The proposed computational brace design method was applied to a cohort of five female potential AIS patients, ranging from 10 to 13 years old, with an average height of 163.6 cm (±11 cm). The subjects were selected because they had to be screened for scoliosis based on previous diagnosis or examination, but subject #2 was not considered an AIS patient after all, and subject #3 would not require brace treatment in clinical practice. As discussed in the definition of the clinical metric in Section 4.1, in our study we use the Cobb angle metric (Safari et al., 2019) to characterize the severity of scoliosis, as it is the standard clinical metric for this purpose. The average Cobb angle measured on the subjects was 17.4° (±8.9°).

In order to proceed with the study, all patients provided oral and written consent according to national Danish guidelines and the Helsinki Declaration, and with approval of the local ethics committee at University Hospital of Hvidovre (No H-17034237). For each subject, we captured biplanar radiographs of the trunk, as they are readily available as part of the regular check-up of scoliotic patients, using a DelftDI D2RS system with fluoroscopic exposure (Wong et al., 2021). Then, we followed the procedure in (Koutras et al., 2022) to morph a template trunk geometry and obtain personalized trunk biomechanical models. Table 1 provides the individual patient data, as well as the complexity of the simulation models, characterized by the number of triangles of the surface mesh (which dominates the cost of contact computations) and the number of tetrahedra of the soft-tissue mesh (which dominates the cost of deformation computations).

**TABLE 1:** Features of the subjects evaluated in our study. The table indicates the severity of the main spine curve (i.e., the Cobb angle) of each subject, as well as the size of the simulation meshes used in our optimization algorithm.

Subject ID	Gender	Age	Height (cm)	Cobb angle (deg)	Soft-tissue tets	Surface tris
1	female	10	157	28.9	12737	682
2	female	11	179	5.5	11781	596
3	female	13	168	13.5	14144	566
4	female	11	150	22.8	8644	548
5	female	13	164	16.5	13459	598
avg (± std dev)	—	11.6 ± 1.3	163.6 ± 11.0	17.4 ± 8.9	12153 ± 2148	598 ± 51

### 5.2 Brace optimization results

**FIGURE 2:**
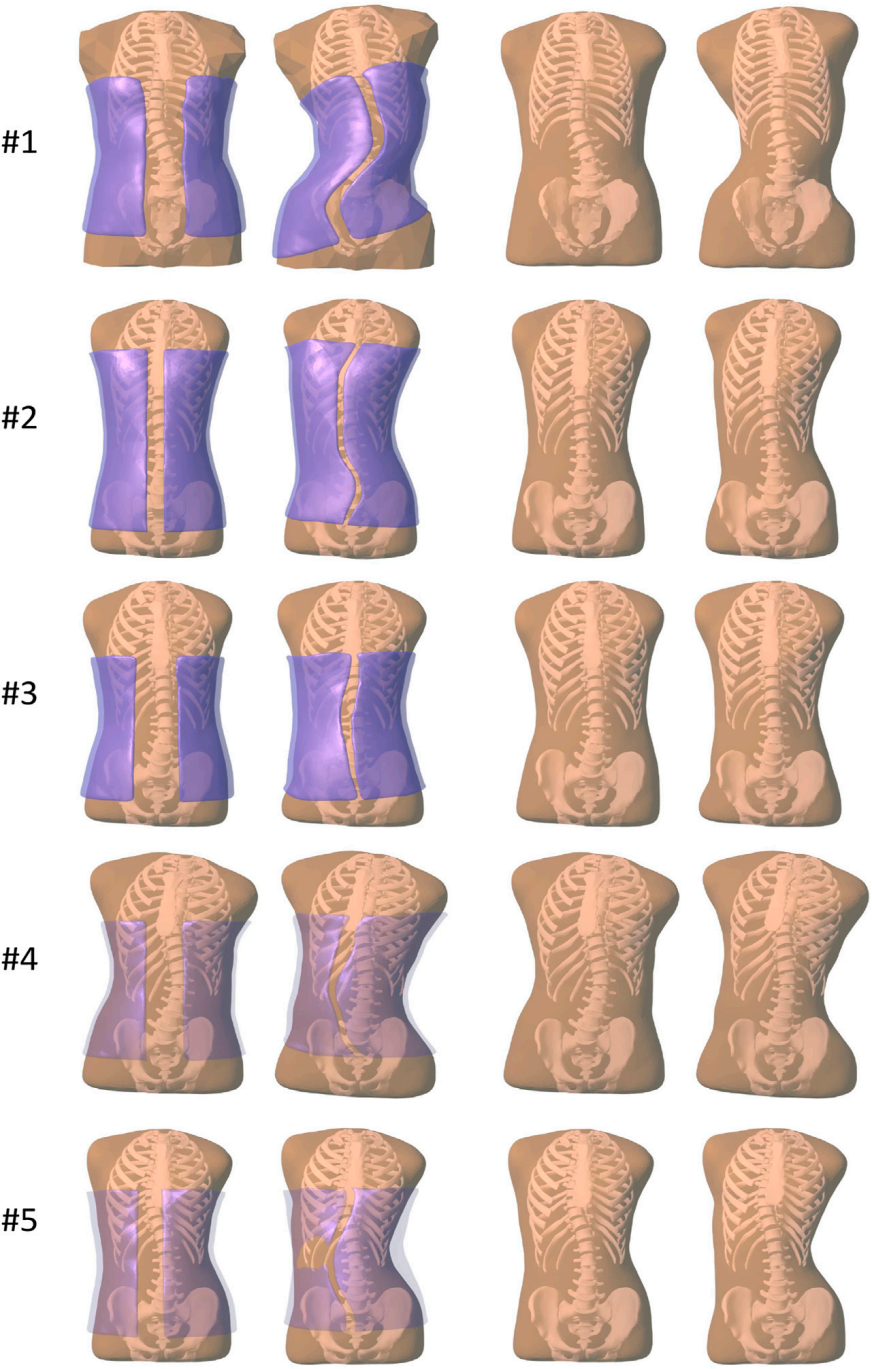
Images of all the subjects evaluated in our study. From left to right, (i) before brace optimization, with the initial brace geometry shown semi-transparent purple, (ii) after brace optimization, with the final brace geometry shown semi-transparent purple, (iii) before brace optimization, just the initial trunk geometry, (iv) after brace optimization, showing the deformed trunk geometry.

Figure 2 illustrates the result of brace optimization for each subject evaluated in the study. The figure shows the trunk geometry and the brace geometry before and after the computational optimization. As evidenced in the images, the optimization algorithm automatically finds brace designs that push on the rib cage at the appropriate location to induce a correction of the spine curvature.

Table 2 compares the Cobb angle before and after applying the scoliosis brace to each of the subjects. Note that we refer to the in-brace correction of Cobb angle; our work has no way of predicting the correction of Cobb angle after brace removal, as this temporal process is not handled by our simulation model. On average, the Cobb angle was reduced by 7.8° (±4.9°), which amounts to a reduction of 45.4% ± 18.9%. Moreover, thanks to the comfort metric described in Section 4.1, this reduction in Cobb angle was achieved under average contact forces of 185.8 N (±29.8 N). None of the subjects reached the limit of 250 N in contact force, indicating a good balance of the clinical and comfort metrics in (Eq. 9). An average reduction of 7.8° in Cobb angle may be considered low with respect to the reduction achieved by real-world braces (in particular in the case of subject #3), and we consider two reasons behind this limitation: 1) The degrees of freedom of our computational braces are currently far fewer than those of real braces; 2) The comfort metric may be too conservative. Both limitations are avenues for further improvement, but they do not challenge the overall methodology.

**TABLE 2:** Quantitative results achieved by optimization-based brace design on each of the test subjects. The table lists the reduction in Cobb angle (in degrees and in percentage of the initial angle), as well as the total force on the subject’s trunk as a measure of comfort.

Subject ID	Cobb before (deg)	Cobb after (deg)	Reduction (deg)	% Reduction	Force (N)
1	28.9	15.7	13.2	45.6	210
2	5.5	2.0	3.5	63.7	152
3	13.5	11.7	1.8	13.8	187
4	22.8	11.8	11.4	50.0	220
5	16.5	7.6	8.9	54.0	160
avg (± std dev)	17.4 ± 8.9	9.7 ± 5.2	7.8 ± 4.9	45.4 ± 18.9	185.8 ± 29.9

Finally, Table 3 summarizes the computational performance of our proposed optimization algorithm. Thanks to differentiable biomechanics and the efficient computation of gradients, the optimizations take 16.4 min on average (±7.3 min). This is in contrast to the average of 94.1 min required by state-of-the-art optimization using finite-difference gradients. The average speed-up across subjects is of 9.2×. This number is particularly affected by the performance of subject #4, for whom the finite-difference version suffered considerably worse convergence. Even removing this outlier case, the average speed-up across subjects is of 4.5×. In our current parameterization of the brace, described in Section 3.2, we use just 6 parameters. With a larger number of parameters, the speed-up of our differentiable biomechanics formulation would be even higher, as the computation of gradients is insensitive to the number of parameters.

**TABLE 3:** Performance of our method, using differentiable biomechanics, compared with state-of-the-art optimization using finite differences (FD) for the computation of gradients. All timings are measured in minutes. We report the number of steps of each optimization, the total time, the time per step, the total speed-up, and the speed-up per step.

Subject ID	Ours			FD			Speed-up	
	Steps	Time	Time/Step	Steps	Time	Time/Step	Total	Per step
1	9	14.3	1.59	14	89.6	6.40	6.3×	4.0×
2	21	16.6	0.79	22	75.6	3.43	4.6×	4.3×
3	17	23.2	1.36	16	67.3	4.20	2.9×	3.1×
4	25	5.2	0.21	29	142.9	4.93	27.4×	23.6×
5	20	22.8	1.14	24	96.4	4.0	4.3×	3.5×
Avg	18.4	16.4	1.02	21.0	94.4	4.60	912×	7.7×

## 6 Discussion

In this paper, we have presented a computational method to design personalized scoliosis Boston braces. The main novelty of the proposed method is to leverage differentiable biomechanics to enable efficient use of numerical optimization methods. Differentiable biomechanics formulates static equilibrium equations as constraints, and evaluates the relationship between deformation state and design variables through implicit differentiation. This relationship is then used within the numerical optimization algorithm to quickly advance toward optimal brace design parameters. We have shown that the differentiable biomechanics formulation provides a large speed-up over state-of-the-art numerical optimization approaches. And this already large speed-up can even increase as the versatility of brace designs grows with more complex parameterizations.

We have demonstrated the use of differentiable biomechanics to design personalized scoliosis braces for five different potential AIS patients. The overall methodology combines personalized trunk simulation models with the differentiable-biomechanics technical contribution. Altogether, we demonstrate that it is possible to design scoliosis braces following a fully computational methodology. The braces balance a clinical metric and a comfort metric, ensuring that they are both effective and practical.

Our study and evaluation are to date limited to synthetic experiments. While the input data belong to real (potential) AIS patients, we have not fabricated and physically tested the brace designs. The contribution of our work is to demonstrate that a computational design methodology is feasible and efficient, but further work is necessary before actual braces are physically tested on real patients. One of the elements that requires further study is the design of the comfort metric. Our current metric considers the total force on the body surface, but this metric is not proven to be suitable or sufficient. Therefore, our work should be complemented with a specific analysis on comfort metrics for scoliosis braces. Another element that requires further study is the accuracy of the overall computational approach, as well as the sensitivity of its various elements. The different computational elements (i.e., the model geometry, the mechanical model, the model parameters, or the weight of the clinical vs comfort metrics) introduce error in the estimated Cobb angle and contact forces. It is necessary to understand the degree of possible error, and thus design a tolerance on the contact forces computed in the simulation. Once the computational model is fully validated, it will be necessary to execute a clinical study concentrated on patient-reported outcome measures, including treatment acceptance and compliance.

In addition to the work necessary for bringing the method to practice, our methodology could also see some improvements on the technical choices. Some of the possible extensions include a richer parameterization of the brace geometry (e.g., using spline surfaces with control points), or personalization of the mechanical parameters of the trunk model. Regarding this last extension, using personalized vs default parameters poses a trade-off between complexity and accuracy. While designing a personalized model would indeed increase accuracy, it is interesting to understand the error produced in practice by a default model.

To conclude, our work has been applied so far only to one type of scoliosis brace, the Boston-type brace, and considering only Cobb angle as clinical metric. It would be interesting to address other types of braces, which would require a different geometric design and a different parameterization. For practical cases, the optimization methodology may even search among different brace types, and find the one that is most convenient for each patient. However, the challenge is two-fold. First, more complex braces require more modeling effort; but this can always be approached by progressively including brace elements of higher impact. Second, some of the design parameters define a continuous parametric space, which can be explored by our current numerical optimization method, but others may define discontinuous or discrete spaces, which require different optimization methods. For instance, optimizing the expansion chambers of Chêneau braces might require topology optimization methods. It would also be interesting to address richer clinical metrics, which are accounted for in the design of braces in practice. One example is the addition of a sagittal-plane metric on top of our current frontal-plane-only metric (e.g., sagittal-plane isostatic balance as a function of lumbopelvic incidence). Our overall approach can be extended to include more advanced multi-objective clinical metrics. For such extension to be possible without critical changes to the overall methodology, the new metrics must be computed from the geometry available in the trunk model, and they must be (locally) continuous and differentiable.

## Data Availability

The data analyzed in this study is subject to the following licenses/restrictions: The dataset was obtained in the context of the MSCA Rainbow project, and use is limited to research within that project. Requests to access these datasets should be directed to miguel.otaduy@urjc.es.
